# Metabolic traits of cancer stem cells

**DOI:** 10.1242/dmm.033464

**Published:** 2018-06-28

**Authors:** Joana Peixoto, Jorge Lima

**Affiliations:** 1 Cancer Signalling and Metabolism Group, Instituto de Investigação e Inovação em Saúde (I3S), Universidade do Porto, 4200-135 Porto, Portugal; 2 Cancer Signalling and Metabolism Group, Institute of Molecular Pathology and Immunology of the University of Porto (Ipatimup), 4200-465 Porto, Portugal; 3 Medical Faculty of the University of Porto, 4200-319 Porto, Portugal; 4 Department of Biochemistry and Molecular Biology, Theodor-Boveri-Institute, Biocenter, 97074 Würzburg, Germany

**Keywords:** Cancer metabolism, Cancer stem cells, Therapy resistance, Tumour heterogeneity

## Abstract

Cancer stem cells are a subpopulation of cells within a tumour believed to confer resistance to standard cancer therapies. Although many studies have addressed the specific mechanisms of tumour recurrence driven by cancer stem cells, cellular metabolism is an often-neglected attribute. The metabolic features of cancer stem cells are still poorly understood, and they thus constitute a promising field in cancer research. The findings published so far point to a distinct metabolic phenotype in cancer stem cells, which might depend on the cancer type, the model system used or even the experimental design, and several controversies still need to be tackled. This Review describes the metabolic phenotype of cancer stem cells by addressing the main metabolic traits in different tumours, including glycolysis and oxidative, glutamine, fatty acid and amino acid metabolism. In the context of these pathways, we also mention the specific alterations in metabolic enzymes and metabolite levels that have a role in the regulation of cancer stemness. Determining the role of metabolism in supporting resistance to therapy driven by cancer stem cells can raise the opportunity for novel therapeutic targets, which might not only eliminate this resistant population, but, more importantly, eradicate the whole tumour in a relapse-free scenario.

## Abstract (precis)

**Summary:** The intrinsic mechanisms that define cancer stem cells, specifically their metabolic properties, are summarized in this Review, in an attempt to point out the benefit of targeting metabolism as a novel therapeutic approach.

## Introduction

Cancer is widely known to be a heterogeneous disease, in which malignant cells communicate with various other cell types, such as endothelial, haematopoietic and stromal cells (see Glossary, Box 1). This complex system within a tumour can influence its own behaviour. Although it is clear that tumour heterogeneity is related to progression, therapy resistance and recurrence, the mechanisms behind these links are still to be uncovered. In this context, a subpopulation of cells within the tumour with the potential for long-term clonal growth and self-renewal capacities – the so-called cancer stem-like cells (CSCs) – has been described as a driver of tumour survival and resistance against commonly used cancer therapies.

**Box 1. Glossary**

**Anaplerotic flux:** the act of replenishing TCA cycle intermediates, as opposed to the use of these molecules as substrates for biosynthetic reactions (cataplerosis).

**Cisplatin:** a platinum-based chemotherapeutic drug used to treat several cancers, including sarcomas, lung cancer, ovarian cancer, lymphomas and germ cell tumours.

**Endothelial cells:** a group of cells that form the surface of blood and lymphatic vessels and function as a barrier between the vessel lumen and the surrounding tissue, being involved in the formation of new blood vessels (angiogenesis).

**Fluorodeoxyglucose-positron emission tomography (FDG-PET):** an imaging technique that uses a glucose analog (FDG) for the visualization of solid cancers. This technique relies on the fact that cancer cells have increased glucose uptake, providing valuable information regarding the localization and size of the tumour for diagnosis, staging and monitoring purposes.

**Haematopoiesis:** the process that yields the formation of blood cells, from a stem cell into a fully differentiated blood cell.

**Haematopoietic cells:** stem cells generally found in the bone marrow that produce all the different blood cell types, by progressing through committed progenitor stages until they fully differentiate into mature cells.

**Hexosamine pathway:** a branch of the glycolysis pathway in which the building blocks for glycosyl side chains of proteins and lipids are produced. It is associated with post-translational modifications, specifically glycosylation.

**Histone demethylase:** an enzyme responsible for removing methyl groups from histones that regulate chromatin architecture.

**Induced pluripotent stem cells:** a type of pluripotent stem cell derived from adult cells by introducing a specific set of reprogramming factors, which induce pluripotency-associated genes.

**Intestinal crypts:** anatomical units where intestinal stem cells are located for the active self-renewal of the intestinal epithelium.

**Leukotrienes:** a family of eicosanoid inflammatory mediators produced by leukocytes**,** mastocytoma cells, macrophages and other cells, in response to immunological stimuli.

**Mammospheres:** round-shaped structures of mammary cells, formed *in vitro* under certain culture conditions to enrich for stem cells.

**Maphosphamide:** the active analogue of the chemotherapeutic drug cyclophosphamide, which is frequently used for *in vitro* experiments.

**Metformin:** a biguanide drug used as a first-line therapy for type 2 diabetes. It is also used as an antitumour agent that affects metabolism by directly inhibiting respiratory chain complex I in the mitochondria.

**Nanog:** a DNA-binding homeobox transcription factor involved in self-renewal and undifferentiation of embryonic stem cells. It is also broadly expressed in human cancers, thus used as a cancer stem cell marker.

**Paclitaxel:** a chemotherapeutic drug that binds to tubulin and inhibits the disassembly of microtubules, ultimately inhibiting cell division.

**Paneth cells:** cells in the intestinal epithelium that are located in the crypts along with intestinal stem cells.

**Pentose phosphate pathway (PPP):** a multi-step metabolic pathway parallel to glycolysis for the oxidation of glucose, which produces NADPH and ribose 5-phosphate that can be used for nucleotide synthesis.

**Satellite muscle cells:** quiescent stem cells of the skeletal muscle that function as a reserve population of cells and proliferate in response to injury.

**Secretome:** the collection of factors released by a cell, including extracellular matrix proteins, transmembrane proteins and vesicle proteins.

**Stemness:** the essential trait of stem cells: their ability to self-renew and differentiate into various committed cells.

**Stromal cells:** a group of connective tissue cells (such as fibroblasts) that support the function of other cells within an organ.

**Temozolomide:** an alkylating chemotherapeutic drug used as treatment for brain tumours.

**^13^C-glucose:** a nonradioactive naturally occurring glucose isotopomer in which all six carbons are ^13^C labelled.

The role of these cells in several cancers has been studied frequently, aiming at disclosing the molecular programs that govern and maintain the stemness (Box 1) of this population. One of these molecular programs encompasses metabolic alterations, which could potentially become important targets for therapies aimed at eliminating this resistant cell population. This Review focuses on the metabolism of cancer stem cells, which is currently an emerging hot topic that researchers need to address further and in a systematic way.

## Stem cells and cancer stem cells

In the late 19th century, Ernst Haeckel used the term stem cell (SC) for the first time to designate the committed cell that gives rise to the germline of an organism. Later in that century, Theodor Boveri and Valentin Häcker pursued and ameliorated the concept of SCs in their embryological studies (Boveri, 1892; Häcker, 1892). In parallel, Artur Pappenheim used the same term to describe the cell that is at the basis of the evolving genealogy of haematopoiesis (Box 1). It was only in the 1960s that James Till, Ernest McCulloch and others provided clear evidence for the existence of a common haematopoietic SC (Till and McCulloch, 1961; Till et al., 1964). These discoveries allowed the establishment of the term SC, which is nowadays used to define a cell capable of proliferating indefinitely and give rise to specialized daughter cells. By raising many questions regarding embryonic development, cellular differentiation and organ maintenance, the role of SCs began to be exploited in disease settings, specifically in cancer (Ramalho-Santos and Willenbring, 2007).

### Intratumour heterogeneity – the hierarchical and stochastic models

The concept of CSCs being identified by the expression of a combination of markers, and the fact that these distinct populations are able to develop a secondary tumour that recapitulates the properties of the primary tumour, was confirmed in several studies (Box 2). Therefore, the CSC model originally postulated a unidirectional hierarchy, where asymmetric and symmetric divisions of CSCs produce the bulk of the tumour to generate differentiated cancer cells and to self-renew the CSC pool, respectively. However, other studies questioned the universality of this hierarchical model, as they showed that cancer cell plasticity often occurs in tumours and that CSCs participate in this process (Nassar and Blanpain, 2016; Prasetyanti and Medema, 2017). For example, stem-like basal and luminal cell populations isolated from human breast cancer cell lines could reversibly convert into distinct cell states to produce the same three subpopulations in similar proportions to the original cell line they were isolated from. However, only the stem-like cells were able to generate tumours upon xenotransplantation, a typical feature of CSCs (Gupta et al., 2011). In a melanoma model, the CSCs expressing the surface marker CD127 were shown to be tumourigenic, but were also able to arise from CD127^−^ progeny, again indicating the reversible phenotype of cancer cells (Quintana et al., 2010; Civenni et al., 2011). Also in melanoma, the expression of the histone demethylase (Box 1) JARID1B (also known as KDM5B) defines a population of slow-cycling cells with tumourigenic potential, i.e. CSCs. Interestingly, JARID1B^−^ cells were shown to re-express the marker upon transplantation, meaning that non-CSCs can rewire to a CSC state (Roesch et al., 2010). CSCs from mouse skin squamous cell carcinoma express CD34 and the transcription factor Sox2 and show plasticity. In this murine model, CD34^−^ and Sox2^−^ cells were both able to form tumours after transplantation, producing CD34- and Sox2-positive and -negative populations in similar proportions to the parental tumour (Schober and Fuchs, 2011; Boumahdi et al., 2014). In contrast, ablation of stem-like cells in a mouse model of glioblastoma was apparently sufficient for tumour growth arrest, as non-CSCs did not replenish a CSC population, which suggests a unidirectional hierarchy in this tumour (Chen et al., 2012). More recent studies, however, suggested that plasticity might occur in glioblastoma under specific conditions and lead to cell reprogramming (Suvà et al., 2014). In fact, non-CSCs can dedifferentiate and acquire the expression of CSC markers in glioblastoma under hypoxic conditions (Wang et al., 2017a) or after treatment with temozolomide (Box 1) (Auffinger et al., 2014) or ionizing radiation (Dahan et al., 2014).

**Box 2. Cancer stem cells**

Tumour heterogeneity is often reflected in the expression of many different histological markers, despite the fact that tumours are believed to arise from a single mutated cell (Nassar and Blanpain, 2016). Advanced technologies, such as fluorescence-activated cell sorting and mouse xenografts assays, allowed studies in haematopoietic stem cells (SCs) that delivered sets of cell-surface markers, which were crucial for defining tumour heterogeneity in acute myeloid leukaemias. Lapidot and colleagues found that only the population of leukaemic cells that were positive for CD34 and negative for CD38 (CD34^+^CD38^−^) could initiate leukaemic engraftment in immune-deficient mice, and that the frequency of these tumour-initiating cells was about one per million cancer cells (Lapidot et al., 1994; Bonnet and Dick, 1997). The term cancer stem-like cells (CSCs) was then coined to define a small population of cells within the cancer that expresses specific markers, and that, when transplanted in immune-deficient mice, is able to recapitulate some of the heterogeneity of the original malignancy (Clevers, 2011; Batlle and Clevers, 2017).

These findings were subsequently recapitulated in studies of solid tumours. Breast cancer was the first human tumour demonstrated to consist of heterogeneous populations of cells, specifically a subpopulation capable of initiating tumour growth in immune-deficient mice. These cells were phenotypically CD44^+^CD24^−/low^ and as few as 100 cells were capable of forming tumours, in contrast to the tens of thousands of cells with other phenotypes that could not reform tumours in mice (Al-Hajj et al., 2003). Moreover, using the same experimental approaches, the specific markers that pinpointed those cells to initiate tumours in immune-deficient mice were identified in several other malignancies, such as pancreatic (Hermann et al., 2007; Li et al., 2007) and colon cancer (O'Brien et al., 2007; Ricci-Vitiani et al., 2007), melanoma (Schatton et al., 2008; Boiko et al., 2010), ovarian (Zhang et al., 2008) and lung cancer (Eramo et al., 2008), and brain tumours (Singh et al., 2004; Chen et al., 2010). However, it became clear that the cell transplantation assay was mainly appropriate for haematological malignancies, in which a defined stem-progenitor hierarchy, with specific markers, was validated in normal haematopoietic SCs and used for the definition of leukaemia SCs (Lapidot et al., 1994; Bonnet and Dick, 1997).

In most solid cancers, this cellular hierarchy and the specific markers for the tissue of origin are still unknown. Commonly used markers, such as CD133, have been extensively used in brain (Singh et al., 2004; Bao et al., 2006) and colon tumours (O'Brien et al., 2007; Ricci-Vitiani et al., 2007) for the characterization of CSCs, but the reproducibility of the findings using this marker has been questioned (Shmelkov et al., 2008; Wang et al., 2008; Chen et al., 2010). Moreover, when sorted for a specific marker, both marker-positive and -negative populations were capable of regenerating the original marker expression of the tumour (Shackleton et al., 2009; Quintana et al., 2010). The fact that intratumour heterogeneity is more pronounced in solid tumours than in leukaemias, together with the lack of specific CSC markers, also limits the universality of this approach for the study of CSCs (Batlle and Clevers, 2017). It should also be noted that transplantation studies are limited to demonstrating that a specific cell population adapts to particular assay conditions, and thus cannot disclose the fate of these cells in their original microenvironment (Clevers, 2011; Nassar and Blanpain, 2016).

In an attempt to overcome the aforementioned limitations, researchers developed genetic lineage-tracing approaches for stem cell studies. The advantage of lineage tracing, as opposed to isolation and transplantation studies, is that knowing which markers are expressed by the cell of interest is not required. When tracing a particular cell type by permanently labelling its progeny with a reporter gene, it is possible to identify cells with SC potential and provide insights into the dynamics of stem and progenitor cells during development, tissue maintenance and repair and, ultimately, their dysregulation in cancer (Kretzschmar and Watt, 2012; Blanpain and Simons, 2013).

Lineage tracing in tumours provided the evidence of a hierarchical organization in solid tumours and therefore consistently proved the existence of CSCs. Lineage tracing of the basal-cell-specific keratin 14 in papillomas demonstrated the long-term survival of a population of cells, which gave rise to large clonal populations within the tumour (Driessens et al., 2012). Another study in a breast cancer mouse model demonstrated that some clones rapidly grew, becoming dominant. CSCs were the origin of these dominant clones and were able to divide into differentiated tumour cells and into new CSCs to sustain tumour growth (Zomer et al., 2013). In colorectal cancer patient-derived organoids, the use of CRISPR-Cas9 gene editing to insert cassettes into the *LGR5* locus – a biomarker of adult stem cells in certain tissues – enabled lineage tracing and revealed that LGR5^+^ cells survive for long time periods, producing progenies that are capable of forming tumours (Cortina et al., 2017; Shimokawa et al., 2017). The formation of large clonal populations from *APC*-deleted Lgr5^+^ cells was observed by lineage tracing, enabling the identification of CSCs in a mouse model of intestinal adenoma (Schepers et al., 2012; Kozar et al., 2013). Furthermore, Chen et al. used a genetically engineered mouse glioma model with a *Nes-ΔTK-IRES-GFP* transgene and identified a population of endogenous cells that were responsible for tumour recurrence. This transgene labelled normal brain SCs in the subventricular zone and also a restricted population of slow-cycling endogenous glioma cells. After tumour proliferation arrest, the nestin^+^ cells re-entered the cell cycle and produced highly proliferative cells that contributed to tumour relapse. Targeted ablation of these cells inhibited tumour progression and recurrence. This study showed that nestin^+^ cells had CSC-like properties, because they demonstrated the capacity of long-term tumour growth and mediated tumour relapse following therapy (Chen et al., 2012).

Overall, the existence of CSCs within the tumour is now accepted as an important feature of cancer and thus, new approaches for studying CSCs are being developed to find novel therapeutic targets.

Thus, in contrast to the unidirectional hierarchic tumour model, a stochastic tumour model with a more fluid hierarchy is now accepted, where cancer cells have the plasticity to dynamically convert from a non-CSC to a CSC phenotype and vice versa in response to appropriate stimuli (Meacham and Morrison, 2013; Medema, 2013). Nevertheless, further studies are required to explore the mechanisms that regulate the reversibility of the CSC phenotype, because this plasticity implies major challenges in the eradication of therapy-resistant cells in cancer, such as CSCs. Specifically, extrinsic factors can influence and alter tumour metabolism, a very important feature of cancer cells that can regulate and maintain cellular fitness in harsh conditions.

## Altered metabolism as a hallmark of cancer

The reprogramming of cellular energy metabolism is a classical feature of cancer, mainly used by cancer cells to sustain their highly proliferative status (Hanahan and Weinberg, 2011). Under aerobic conditions, normal nonproliferating cells use glycolysis in the cytoplasm to form pyruvate, which is then oxidized in mitochondrial oxidative phosphorylation (OXPHOS) to generate energy in the form of adenosine triphosphate (ATP). Under anaerobic conditions, glycolysis-derived pyruvate is mainly directed to lactate production. In contrast, cancer cells rely more on glycolysis for energy production even in the presence of oxygen, a phenomenon first observed by Otto Warburg and termed ‘aerobic glycolysis’ or ‘the Warburg effect’ (Warburg, 1956a,b). This metabolic adaptation, although generating ATP more rapidly, is far less efficient than OXPHOS, resulting in abnormally high glucose uptake to sustain ATP production. The avidity of cancer cells for glucose, mainly mediated by the upregulation of glucose transporter 1 (GLUT1; also known as SLC2A1), contributed to the development of fluorodeoxyglucose-positron emission tomography (FDG-PET; Box 1) techniques for cancer detection and monitoring (Vander Heiden et al., 2009).

As demonstrated in several studies discussed below, increased glycolysis allows the production of several metabolic intermediates that can feed alternative biosynthetic pathways to generate macromolecules, such as nucleosides, amino acids and lipids, which can then be used as building blocks to support the high proliferation and division rates of cancer cells, conferring a selective advantage.

The factors underlying the metabolic alterations of cancer cells are the subject of intense study. Oncogenes or tumour suppressors, but also the tumour microenvironment (TME), can reprogram cancer metabolism by directly regulating specific metabolic enzymes. Oncogenic mutations in phosphatidylinositol 3-kinases (PI3K; also known as PIK3CA) promote metabolic reprogramming by enhancing AKT [also known as AKT1 or protein kinase B (PKB)] signalling, which, in turn, drives glycolytic metabolism by increasing cellular glucose uptake and inducing the activation of phosphofructokinase 1 (PFK1) (Deprez et al., 1997; Elstrom et al., 2004; Manning and Cantley, 2007). In addition, AKT stimulates the mammalian target of rapamycin (mTOR) pathway, which also promotes glycolysis and the pentose phosphate pathway (PPP; Box 1) through the regulation of hypoxia-inducible factors (HIFs) (Düvel et al., 2010). Similarly, Myc dysregulation in cancer is associated with inducing the expression of glycolytic genes, causing a shift to glucose consumption, as well as biomolecule production via nucleotide and lipid synthesis (Shim et al., 1997; Osthus et al., 2000; Nikiforov et al., 2002; Kim et al., 2007; Morrish et al., 2010). Mutations in the small GTPase RAS subfamily have also been associated with metabolic reprogramming towards glycolysis, the hexosamine pathway (Box 1) and the PPP, in a process mediated by the PI3K/AKT/mTOR axis or by MYC (Ramanathan et al., 2005; Gaglio et al., 2011; Ying et al., 2012). The tumour suppressor p53 (also known as TP53) inhibits glucose transporters and triggers the upregulation of TP53-induced glycolysis regulator (TIGAR), causing a decrease in fructose 2,6-biphosphate levels and thus inhibition of PFK1 (Bensaad et al., 2006). In addition, p53 stimulates the expression of the gene encoding the synthesis of cytochrome c oxidase protein (SCO2), a subunit of complex IV of the electron transport chain required for assembly of the cytochrome c oxidase (COX) complex (Matoba et al., 2006). Thus, loss of p53 promotes a shift in ATP production from OXPHOS to glycolysis, but renders cancer cells more sensitive to metabolic stress (Matoba et al., 2006). The involvement of the TME in metabolic reprogramming is mainly mediated by HIF-1α and HIF-2α. The upregulation of HIF-1α and HIF-2α under hypoxic conditions is one of the mechanisms by which tumour cells can trigger the switch from OXPHOS to glycolysis. Specifically, HIF-1α induces the expression of GLUT1, and upregulates glycolytic enzymes and lactate dehydrogenase A (LDHA), with concomitant activation of pyruvate dehydrogenase kinase 1 (PDK1), a negative regulator of pyruvate dehydrogenase (PDH) (Semenza et al., 1994; Kim et al., 2006; Papandreou et al., 2006).

In addition to glucose metabolic changes, increased glutamine metabolism is a common feature of cancer cells (Medina et al., 1992; Souba, 1993). Glutamine is essential in proliferating tumour cells, providing defence mechanisms against oxidative stress, synthesising macromolecules when glucose metabolism is not sufficient and, ultimately, fuelling cellular bioenergetics (Deberardinis et al., 2008). Furthermore, oncogenes such as *MYC* can influence glutamine metabolism, as they do for glucose metabolism (Wise et al., 2008). MYC stimulates the expression of surface transporters to drive glutamine metabolism and can also regulate glutaminase (GLS) by supressing the microRNAs responsible for preventing *GLS* translation (Gao et al., 2009). Glutamine dependency can also modulate the signal transduction pathways that contribute to tumour growth. For example in HeLa cells, glutamine excess leads to a bidirectional transport of this amino acid through membrane transporters, accompanied by an import of other essential amino acids (Nicklin et al., 2009). This mechanism subsequently activates mTORC1, stimulating cell growth and supressing catabolism and autophagy. ERK (also known as EPHB2) signalling is another example of glutamine-dependent activation found in intestinal epithelial cells (Rhoads et al., 1997; Larson et al., 2007), and in melanoma (Pollock et al., 2003; Ohtani et al., 2008) and glioma cells (Arcella et al., 2005).

A major outcome of both glucose and glutamine metabolism is the production of citrate to support cellular bioenergetics and to produce biomass, namely nucleic acids, proteins and lipids, necessary for cell proliferation. The metabolic fate of citrate produced by these two main metabolic processes is defined by its subcellular localization: mitochondrial citrate is shuttled to the tricarboxylic acid (TCA) cycle, whereas cytoplasmic citrate feeds fatty acid synthesis (Currie et al., 2013).

Lipid metabolism is another major source of metabolic intermediates and energy for processes involved in cell transformation and tumour progression (Santos and Schulze, 2012). Cancer cells can fulfil their strong avidity for lipids either by increasing exogenous lipid uptake or endogenous production through *de novo* synthesis (Medes et al., 1953; Ookhtens et al., 1984). Lipid synthesis requires several steps to convert citrate into bioactive fatty acids, which are undertaken by ATP citrate lyase (ACLY), acetyl-CoA carboxylase (ACC), fatty acid synthase (FASN) and acyl-CoA synthetase (ACS). Additionally, fatty acid biosynthesis is mainly controlled by sterol regulatory element-binding proteins (SREBPs), a family of transcription factors that bind to sterol regulatory elements and some E-box sequences in the promoters of target genes (Röhrig and Schulze, 2016). SREBP1 (also known as SREBF1) can be activated through an AKT-mTORC1 signalling axis, thus promoting lipid synthesis and cell growth (Porstmann et al., 2008). In glioblastomas, the presence of a constitutively active mutant epithelial growth factor receptor (EGFR), the EGFR variant III (EGFRvIII), functions as an enhancer of activated SREBP1 in the nucleus, being correlated with increased levels of FASN and ACC (Guo et al., 2009). In glioblastomas that do not carry EGFR mutations, but instead have AKT pathway activation, silencing of SREBP1 or SREBP2 prevented xenograft growth, confirming the importance of fatty acid metabolism in tumour maintenance (Griffiths et al., 2013; Williams et al., 2013).

Furthermore, stearoyl-CoA desaturase (SCD), an enzyme involved in fatty acid biosynthesis and a target gene of SREBP1, is overexpressed in several human cancers (Li et al., 1994; Falvella et al., 2002; Fritz et al., 2010). Silencing of SCD inhibits lipid synthesis and activates AMP-activated protein kinase (AMPK; also known as PRKAA2), which in turn increases β-oxidation of fatty acids (Dobrzyn et al., 2004), leading to tumour size reduction in xenograft models of liver (Budhu et al., 2013), lung (Scaglia and Igal, 2008) and stomach (Roongta et al., 2011) cancers, as well as to inhibition of prostate cancer progression in mice (Fritz et al., 2010).

As a disease that requires building blocks for cell proliferation and survival, cancer is characterized by metabolic remodelling meant for accumulating metabolic intermediates, which are then used as a source of biomass. In the setting of SCs and CSCs, such altered metabolism is also able to shape and regulate the fate and function of these specific cells.

## Metabolic phenotypes of SCs and CSCs

In general, normal tissue hierarchy holds a metabolic phenotype where multipotent SCs primarily perform glycolysis, while differentiated cells are more reliant on OXPHOS. The first studies on the metabolic phenotype of SCs, again using haematopoietic cells, revealed that SCs reside in hypoxic niches and use mainly glycolysis (Parmar et al., 2007; Suda et al., 2011). Additionally, they have fewer and less mature mitochondria than differentiated cells, resulting in a lower production of reactive oxygen species (ROS) (Jang and Sharkis, 2007; Prigione et al., 2010). In contrast, the differentiated progeny of haematopoietic SCs shift to OXPHOS and increased production of ROS. Although OXPHOS is crucial for the energy demands of complex tissues, SCs might avoid this metabolic phenotype because of the resulting high levels of ROS, which in turn could lead to SC dysfunction (Simsek et al., 2010; Suda et al., 2011; Maryanovich et al., 2015). Therefore, the quiescent state of adult SCs can serve as a protective mechanism against oxidative stress-related damage, ensuring the infinite self-renewal capacity of these cells (Folmes et al., 2012). Furthermore, cellular metabolism can actually control stemness. When reprogramming somatic cells into induced pluripotent SCs (Box 1), the upregulation of glycolytic genes preceded the expression of pluripotency markers, revealing that the metabolic switch from OXPHOS to glycolysis is an early event during SC reprogramming (Folmes et al., 2011; Panopoulos et al., 2012). However, this metabolic pattern can differ between adult SC populations. For example, in intestinal crypts (Box 1), Lgr5^+^ SCs have increased OXPHOS, whereas Paneth cells (Box 1) preferentially use glycolysis. Paneth cells regulate the renewal of Lgr5^+^ SCs by producing lactate for the oxidative metabolism of the SCs (Rodríguez-Colman et al., 2017). Apparently, high ROS levels are not harmful to intestinal SCs, but rather induce their differentiation (Yilmaz et al., 2012; Rodríguez-Colman et al., 2017). Another example is satellite muscle cells (Box 1), which are localized in aerobic niches and use mainly OXPHOS; in contrast, committed progenies of these SCs undergo epigenetic reprogramming consistent with a shift to glycolytic metabolism (Ryall et al., 2015).

The metabolic phenotype of CSCs has been studied over the past few years and, contrarily to what was hypothesized, CSCs do not recapitulate the metabolic pattern of adult SCs. In fact, CSCs can primarily rely either on glycolysis or on OXPHOS, mainly depending on the tumour type and TME stimuli that trigger cell plasticity and metabolic reprogramming (Sancho et al., 2016).

### Glycolysis

Glycolysis is an oxygen-independent metabolic pathway that occurs in the cytosol, generating ATP from the conversion of glucose into pyruvate. It consists of three main reactions: phosphorylation of glucose by hexokinase to form glucose 6-phosphate and subsequently fructose 1,6-biphosphate (F1,6P); cleavage of F1,6P into two three-carbon products (glyceraldehyde 3-phosphate and dihydroxyacetone phosphate); and oxidation of these three-carbon products to pyruvate, with ATP production.

**Fig. 1.**
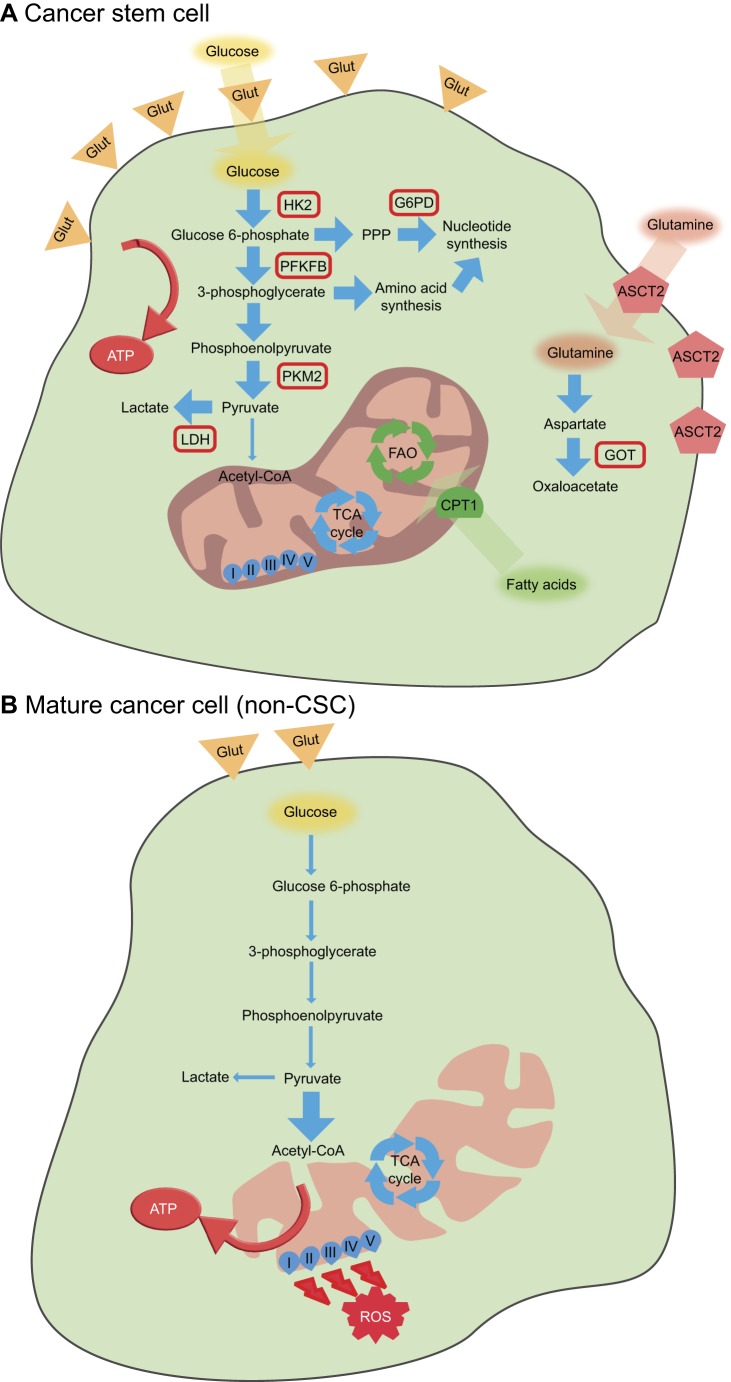
**General metabolic features of cancer stem cells and mature cancer cells (non-CSCs).** (A) Cancer stem cells tend to rely more on glycolysis for ATP synthesis, with overexpression of the glucose transporters GLUT1 and GLUT3, and increased expression of hexokinase 2 (HK2), 6-phosphofructo-2-kinase/fructose-2,6-biphosphatase (PFKFB), pyruvate kinase isozyme M2 (PKM2) and lactate dehydrogenase (LDH). Nucleotide biosynthesis is often increased in cancer stem cells owing to overexpression of glucose-6-phosphate dehydrogenase (G6PD) and amino acid synthesis. Glutamine uptake and metabolization to oxaloacetate, together with fatty acid oxidation, also appear to be important mechanisms in cancer stem cells. (B) In contrast, mature cancer cells tend to rely more on OXPHOS for adenosine triphosphate (ATP) production, leading to increased levels of reactive oxygen species (ROS); these cells show low levels of glycolysis and nucleotide synthesis, although this can vary. ASCT2, alanine, serine, cysteine-preferring transporter 2; CPT1, carnitine-dependent transporter 1; FAO, fatty acid oxidation; Glut, glucose transporter (GLUT1 or GLUT3); GOT, glutamate-oxaloacetate transaminase; PPP, pentose phosphate pathway; TCA, tricarboxylic acid.

As discussed below, CSCs usually have significantly increased glucose uptake and lactate production, together with a decrease in mitochondrial respiration, when compared with their mature non-CSC counterparts (Fig. 1A,B).

In breast cancer cell lines, a switch from mitochondrial respiration to glycolysis decreases the levels of ROS, a mechanism that is essential for the maintenance of stemness in CD44^+^CD24^−^EPCAM^+^ cells (Dong et al., 2013). Besides, key glycolysis enzymes, such as pyruvate kinase M2 (PKM2; also known as PKM), LDH and glucose-6-phosphate dehydrogenase (G6PDH) have increased activity in breast CSCs, while treatment with 2-deoxyglucose (2DG), a glucose analogue that inhibits hexokinase 2 (HK2), preferentially decreases the proliferation of these cells compared with mature cancer cells, showing that glycolysis is essential for breast CSCs (Ciavardelli et al., 2014). CSCs isolated from human glioblastoma xenografts also have increased glycolysis and low mitochondrial respiration, with a downregulation of the succinate dehydrogenase subunit B (SDHB) and subsequent mitochondrial dysfunction, leading to increased electron leakage and ROS production. Through an ROS-mediated mechanism, the basal levels of HIF-1α and HIF-2α increase, promoting glycolysis by upregulating GLUT1 and HK2 in these human glioblastoma SCs. Furthermore, hypoxia renders glioblastoma SCs resistant to conventional anticancer agents and sensitive to glycolytic inhibition, suggesting that they have a preference for hypoxic environments and a glycolytic metabolism to maintain their stemness (Zhou et al., 2011). Genes involved in glycolysis, namely *PKM2* and 6-phosphofructo-2-kinase/fructose-2,6-biphosphatase 4 (*PFKFB4*), were also identified as stemness regulators in glioma SCs (Morfouace et al., 2014) (Fig. 1A). On one hand, experimental knockdown of PFKFB4 caused a reduction in lactate and ATP production, inducing apoptosis of glioblastoma SCs. On the other hand, overexpression of this enzyme was associated with shorter survival of glioblastoma patients (Goidts et al., 2012). In nutrient-deprived conditions, glioblastoma SCs also showed an upregulation of GLUT3 (also known as SLC2A3), a transporter with higher affinity for glucose than GLUT1, in order to preserve glycolysis and maintain stemness (Flavahan et al., 2013). Mao et al. found that two distinct tumour-derived glioblastoma SC subtypes – proneural and mesenchymal glioblastoma SCs – were prominently correlated with the clinically recognized proneural and mesenchymal subtypes of glioblastoma and had distinct dysregulated signalling pathways (Mao et al., 2013). Mesenchymal glioblastoma SCs highly expressed the aldehyde dehydrogenase (ALDH) family, especially the enzyme ALDH1A3, which is involved in glycolysis, among other functions. Inhibition of ALDH1A3 attenuates the growth of this subtype of CSCs, but not of the proneural subtype, suggesting that different CSC populations can have distinct stemness-regulating metabolic pathways (Mao et al., 2013). To further highlight their metabolic variability, glioblastoma SCs were also reported to consume less glucose and produce less lactate, while having higher ATP levels, when compared with differentiated cancer cells (Vlashi et al., 2011). In this study, inhibition of either glycolysis or mitochondrial respiration in CSCs had minimal effect on energy production and only the combined inhibition of both pathways was able to deplete intracellular ATP levels. These CSCs revealed metabolic plasticity features, indicating that targeting specific metabolic pathways individually might not be sufficient to eradicate glioblastoma SCs (Vlashi et al., 2011).

In a mouse model of hepatocellular carcinoma, CSCs expressing Nanog (Box 1) have increased glycolytic activity and fatty acid oxidation (FAO), decreased mitochondrial respiration – owing to cytochrome C oxidase subunit 6A2 (Cox6a2) repression, and inhibition of ROS generation (Fig. 1A), suggesting that a defined metabolic pattern regulates stemness in this model (Chen et al., 2016).

Emmink et al. compared the secretome (Box 1) of CSCs and differentiated cells from colorectal tumours, and observed that CSCs have enriched levels of proteins involved in glycolysis and antioxidant pathways. Furthermore, CSCs secreted high levels of ALDH, which is implicated in the detoxification from anticancer drugs, such as maphosphamide (Box 1). By secreting this drug-detoxifying enzyme, CSCs could not only promote self-preservation, but also protect the differentiated mature cancer cells in their vicinity. Briefly, colorectal CSCs had a survival and antioxidant signature, both of which contributed to therapy resistance (Emmink et al., 2013).

In a metabolic study that used ^13^C-glucose (Box 1) in ovarian cancer, CSCs showed an enrichment in glycolysis, the PPP and *de novo* fatty acid synthesis, while having a decrease in mitochondrial respiration and anaplerotic flux (Box 1, Fig. 1A); in contrast, mature cancer cells showed increased mitochondrial respiration and higher anaplerotic flux (Fig. 1B). Additionally, cisplatin treatment (Box 1) resulted in higher survival of ovarian CSCs in comparison with mature cancer cells. Thus, the authors concluded that this metabolic phenotype of CSCs might contribute to more aggressive tumours and confer increased therapy resistance (Liao et al., 2014).

### Mitochondrial respiration

Mitochondrial respiration comprises a series of chemical reactions that occur in the mitochondria, for generating ATP in the presence of oxygen. This metabolic pathway is far more efficient in energy production than glycolysis, generating 36 molecules of ATP per molecule of glucose, as opposed to two molecules of ATP produced in glycolysis.

As briefly mentioned in the previous section, there are reports claiming that CSCs consume less glucose, produce less lactate and are mainly OXPHOS dependent and less glycolytic than their differentiated counterparts. Leukaemia SCs, although showing low levels of ROS, have an overactive BCL-2-dependent OXPHOS; indeed, inhibiting BCL-2 reduces OXPHOS and can therefore eradicate CSCs (Lagadinou et al., 2013). Likewise, breast cancer cell lines with higher mitochondrial mass and activity were enriched in CSC markers, had a higher efficiency in forming mammospheres (Box 1), had increased tumour initiation capacity in murine xenografts and were resistant to paclitaxel treatment. The authors thus considered mitochondrial mass a potential metabolic biomarker of CSCs (Farnie et al., 2015; Lamb et al., 2015). In ovarian cancer, CSCs also had an OXPHOS-dominated metabolic profile with high ROS production and increased mitochondrial membrane potential (Pastò et al., 2014). Pancreatic ductal adenocarcinoma (PDAC) is another example of OXPHOS-dependent metabolism in CD133 (also known as PROM1)-expressing CSCs, when comparing with mature cancer cells. In this model of patient-derived xenografts, the transcription factor peroxisome proliferator-activated receptor-gamma coactivator (PGC-1α), a regulator of mitochondrial biogenesis, was essential for the OXPHOS phenotype of CSCs, and also for their self-renewal and *in vivo* tumorigenic capacities. In contrast, differentiated PDAC cells have a MYC-driven glycolytic phenotype, in which MYC overexpression negatively controls PGC-1α expression and inhibits stemness (Sancho et al., 2015).

Glycolysis and OXPHOS are not necessarily mutually exclusive. Indeed, in a study using breast CSCs, Vlashi et al. reported higher glucose consumption, concomitant with lower lactate and higher ATP production, and with a consistent increase in mitochondrial capacity and activity, compared with the differentiated progeny (Vlashi et al., 2014).

### Glutamine metabolism

Glutamine metabolism is an anabolic process that produces macromolecules with lower energetic potential (Zheng, 2012). Glutamine can enter cells through the alanine, serine, cysteine-preferring transporter 2 (ASCT2; also known as SLC1A5) and then be hydrolyzed to glutamate and ammonia through the action of GLS. Glutamate, on one hand, can be combined with cysteine and glycine to form reduced glutathione (GSH), which is a major antioxidant that regulates oxidative stress (Estrela et al., 2006); on the other hand, glutamate can be converted into α-ketoglutarate (αKG) to provide TCA cycle intermediates and, ultimately, energy production. This process is relevant for cells that lack citrate production owing to inefficient usage of glucose, constituting an alternative pathway for a truncated TCA cycle (DeBerardinis and Cheng, 2010).

In human haematopoietic SCs, the differentiation to the erythroid lineage completely depends on glutamine metabolism. Even in the presence of erythropoietin, glutamine-depleted haematopoietic SCs were diverted towards a myelomonocytic differentiation. Knockdown of ASCT2 decreased glutamine uptake and inhibited erythroid differentiation. Additionally, the authors demonstrated that the commitment to an erythroid state was not restored by solely feeding the TCA cycle with cell-permeable αKG, but rather it depended on nucleotides produced by glutamine metabolism (Oburoglu et al., 2014).

In colorectal cancer cell lines, glutamine metabolism was found to regulate the sensitivity of CSCs to metformin (Box 1) through the AMPK-mTOR pathway. In the presence of glutamine, CSCs from the SW620 cell line showed resistance to metformin, while in the absence of glutamine, these CSCs showed activation of AMPK, suppression of mTOR and became sensitive to metformin treatment. In contrast, CSCs from the HT29 cell line were sensitive to metformin, because they have an activated AMPK pathway. Nevertheless, inhibition of glutamine metabolism in these cells increased the CSC-suppressive effect of metformin. CSCs from both cell lines showed higher expression of ASCT2 in comparison with mature cancer cells, and knockdown of ASCT2 significantly decreased the proportion of CSCs (CD133^+^ CD44^+^) in comparison with control small interfering RNA (Fig. 1A). Thus, glutamine metabolism plays an important role in regulating the different responses of CSCs to metformin (Kim et al., 2018).

Another study using PDAC cells demonstrated that a novel noncanonical glutamine pathway is essential for tumour growth and oxidative stress balance in CSCs (Li et al., 2015). Here, glutamine deprivation significantly decreased the expression of stemness markers and the self-renewal potential, and increased intracellular ROS levels, inducing apoptosis. When using glutamine-free medium supplemented with oxaloacetate, the product of the noncanonical glutamine metabolism, these effects were rescued; in contrast, the product of the canonical glutamine metabolism, αKG, did not rescue these effects on CSCs. Additionally, CSCs showed increased expression of GLS and glutamate-oxaloacetate transaminases (GOT1 and GOT2), the latter converting glutamine-derived aspartate to oxaloacetate (Fig. 1A). Inhibition of this pathway sensitized CSCs to radiation, showing that the combination of a glutamine metabolism inhibitor with radiotherapy might be a suitable therapy for PDAC (Li et al., 2015).

### Fatty acid metabolism

Besides using glucose as a fuel for anabolic processes, cells can derive their energy from fatty acid metabolism. This is essentially controlled by: (1) fatty acid synthesis (FAS), an anabolic process that converts acetyl-CoA to malonyl-CoA and is required for cell growth and proliferation; and (2) fatty acid oxidation (FAO), a catabolic process that breaks down fatty acids to generate acetyl-CoA for anaplerosis, as well as NADH for the production of ATP (Carracedo et al., 2013).

Haematopoietic SCs are maintained through an FAO pathway downstream of promyelocytic leukaemia protein (PML) and peroxisome proliferator-activated receptor δ (PPARδ). This PML–PPAR–FAO axis is essential for haematopoietic SCs, as it controls the asymmetric division and the fate of these cells (Ito et al., 2008, 2012). Indeed, the SC population can be exhausted by pharmacological or genetic inhibition of any component of this pathway, suggesting that FAO is crucial for stemness (Ito et al., 2012). Another FAO-associated protein, liver kinase B1 (LKB1; also known as STK11), was also found to be essential for haematopoietic SC maintenance (Gan et al., 2010). LKB1 activates and phosphorylates AMPK in response to a decline in the ATP/adenosine monophosphate (AMP) ratio; in turn, AMPK phosphorylates key regulatory proteins involved in fatty acid metabolism to restore ATP levels (Gan et al., 2010; Gurumurthy et al., 2010). Deletion of *Lkb1* in mice causes rapid depletion of the haematopoietic SC pool, while also inducing alterations in lipid metabolism, depletion of cellular ATP and mitochondrial defects in *Lkb1*-deficient bone marrow cells (Gan et al., 2010; Gurumurthy et al., 2010; Nakada et al., 2010).

Adult murine neuronal stem and progenitor cells also have increased activity of fatty acid synthase (Fasn), a key enzyme for *de novo* lipogenesis, in comparison with differentiated progenies (Knobloch et al., 2013). Fasn is required for the proliferation of progenitor cells, while quiescent, nonproliferating SCs tended to shift from FAS towards FAO. Low proliferating SCs selectively express the thyroid hormone-responsive protein Spot14 (also known as Thrsp), which reduces lipid synthesis and acts as a molecular brake on Fasn-dependent lipogenesis. Thus, neurogenesis is sustained through a tight regulation of fatty acid metabolism, where Fasn and Spot14 play a major role in the regulation of malonyl-CoA levels for the generation of complex fatty acids, which in turn regulate the more quiescent or committed state of the neuronal cells (Knobloch et al., 2013).

Similarly to haematopoietic SCs, quiescent leukaemia-initiating CSCs were also shown to be regulated by FAO. Recent reports revealed that a subpopulation of leukaemia SCs, expressing the fatty acid transporter CD36, can reside in gonadal adipose tissue niches to induce lipolysis and fuel FAO, leading to chemotherapy resistance (Ye et al., 2016). CD36^+^ cells are also present in oral squamous cell carcinomas and comprise a population of slow-cycling cells expressing the stem cell marker CD44, while also expressing high levels of lipid metabolism genes and being associated with metastasis (Pascual et al., 2017). CD36^+^ cells were additionally found in other tumours, namely in melanoma and breast cancer, where they also associate with metastatic potential (Pascual et al., 2017). These studies suggest that a subset of highly aggressive CSCs obtain their energy through FAO, thus revealing a specific metabolic profile required during metastasis that might be a potential target for the eradication of CSCs. Furthermore, in hepatocellular carcinoma, Nanog induces a metabolic reprogramming of CSCs: a decrease in mitochondrial respiration and an enhanced reliance on glycolysis and, additionally, an upregulation of FAO genes to support the self-renewal of these cells (Chen et al., 2016) (Fig. 1A). In contrast, *de novo* lipid synthesis is increased in glioma CSCs compared with mature glioma cells, owing to high Fasn expression. Fasn inhibition reduces stem cell marker expression while increasing differentiation markers and decreasing the proliferation and migration of CSCs (Yasumoto et al., 2016) (Fig. 1A). Other lipid metabolism enzymes, such as arachidonic acid 5-lipoxygenase (ALOX5), which is involved in the synthesis of leukotrienes (Box 1) from arachidonic acid, and acyl-CoA synthetase very-long-chain 3 (ACSVL3; also known as SLC27A3), a key enzyme in fatty acid activation for the formation of fatty acyl-CoA, were also shown to support glioblastoma CSCs self-renewal and to induce tumour xenograft formation (Wang et al., 2011; Sun et al., 2014). Finally, colorectal CSCs have high levels of lipid droplets, which correlate with the expression of stem cell markers. CSCs with more lipid droplets showed a higher tumourigenic capacity upon xenotransplantation, suggesting that lipid metabolism can mediate stemness in colorectal cancer (Tirinato et al., 2015).

### Other metabolic features

Mutations in genes that encode metabolic enzymes have revealed yet another mechanism of cancer stemness regulation by metabolic reprogramming. In leukaemia, mutations in isocitrate dehydrogenase 1 and 2 (*IDH1* and *IDH2*, respectively) were specifically associated with CSC regulation. These mutations alter the normal IDH1/2-mediated conversion of isocitrate to αKG into an aberrant conversion of αKG to the analogue, 2-hydroxyglutarate (2-HG). This metabolite accumulates intracellularly and inhibits tet methylcytosine dioxygenase 2 (TET2) function by competing with its cofactor αKG. In the presence of mutant *IDH1*/*2* or *TET2* depletion, the self-renewal potential of haematopoietic SCs increases and differentiation is impaired, which suggests a pro-leukaemic phenotype (Figueroa et al., 2010; Cimmino et al., 2011; Kats et al., 2014).

Purine synthesis is another metabolic mechanism that controls stemness in brain CSCs. Wang et al. observed that CSCs show upregulation of enzymes involved in purine synthesis for the production of purine nucleotides that serve as building blocks for DNA and RNA (Wang et al., 2017b). These include the enzymes phosphoribosyl pyrophosphate synthetase 1 (PRPS1) and phosphoribosyl pyrophosphate amidotransferase (PPAT) to synthesize inosine monophosphate (IMP), adenylosuccinate lyase (ADSL) and adenylosuccinate synthase (ADSS) to synthesize AMP, and guanine monophosphate synthase (GMPS) and IMP dehydrogenase 1 (IMPDH1) to synthesize guanosine monophosphate (GMP). Genetic perturbations of these enzymes caused a decrease in CSC growth and maintenance and abrogated tumour formation in immunodeficient and immunocompetent mouse models, by depleting the intracellular pools of purine nucleotides. In contrast, targeting purine biosynthesis did not affect differentiated glioblastoma cells, which collectively supports the selective dependence of brain CSCs on the purine synthesis pathway (Wang et al., 2017b).

Lysine catabolism was also found to be essential to promote the self-renewal of colorectal CSCs and induce liver metastasis. Colorectal CSCs expressing the thrombopoietin-binding receptor CD110 (also known as MPL) signal through thrombopoietin by activating lysine degradation. This generates acetyl-CoA, which is used for the acetylation of the LDL receptor-related protein 6 (LRP6). This acetylation in turn recruits casein kinases that phosphorylate LRP6 for the activation of WNT protein signalling and regulation of CD110^+^ CSC self-renewal. Furthermore, lysine catabolism in these cells promoted the generation of glutamate, which enhances cysteine uptake and GSH synthesis. Thus, CD110^+^ CSCs are able to modulate their redox status by lysine catabolism, promoting self-renewal, drug-resistance and liver metastasis (Wu et al., 2015).

Normal SCs seem to have a more consistent metabolic phenotype and control of their molecular pathways (Ito and Suda, 2014). As discussed in this section, CSCs, although lacking a common metabolic pattern across cancer types, clearly have a distinctive metabolic phenotype compared with their mature cancer cell counterparts. The number of publications addressing the metabolism of CSCs is still small, and discrepancies, such as different environmental stimuli in the experimental setting, might explain the contradictory results. As an example, many of the studies discussed in this Review favour glycolysis by growing cells in high glucose and high oxygen culture conditions that fail to recapitulate tissue homeostasis and TME conditions *in vivo*. Indeed, the TME is an important factor for cellular metabolism, as it creates a symbiotic system where, for example, highly glycolytic stromal cells generate metabolic products that can be used by cancer cells; these, in turn, shift their metabolism towards OXPHOS and can potentially trigger a reprogramming to more stem-like states (Pavlides et al., 2009; Migneco et al., 2010; Martinez-Outschoorn et al., 2011; Nakajima and Van Houten, 2013; Davidson et al., 2016). Such organization cannot be reproduced when using established cancer cell lines in *in vitro* systems that lack a suitable TME, resulting in inconsistent findings and major differences concerning the metabolic phenotypes in *in vitro* and *in vivo* settings. Importantly, the very definition of CSCs and the experimental designs for their isolation and characterization vary across studies, which also contributes to the inconsistency of results.

Although these findings still lack robustness and further validations, the specific metabolic features of CSCs have been tested as potential therapeutic targets and should be taken into account for future cancer therapies.

## Metabolism as a therapeutic target for CSCs

Cancer cell plasticity and the acquisition of a quiescent state are thought to be important drivers of drug resistance. Actually, several findings support the fact that residual dormant clones, which resist the antiproliferative chemotherapeutic treatment, can become dominant and cause tumour relapse (Chen et al., 2012; Kreso et al., 2013; Kurtova et al., 2015; Liau et al., 2017). Along with the emergent reports aiming at characterizing the molecular mechanisms that govern stemness in cancer, several therapeutic approaches have been developed and tested for the elimination of CSCs. However, no anti-CSC therapy has shown sufficient effectiveness in order to be approved for clinical use. Thus, therapies targeting the metabolic networks that mediate cancer cell stemness could be an innovative and efficient strategy to target this cell population.

**Fig. 2.**
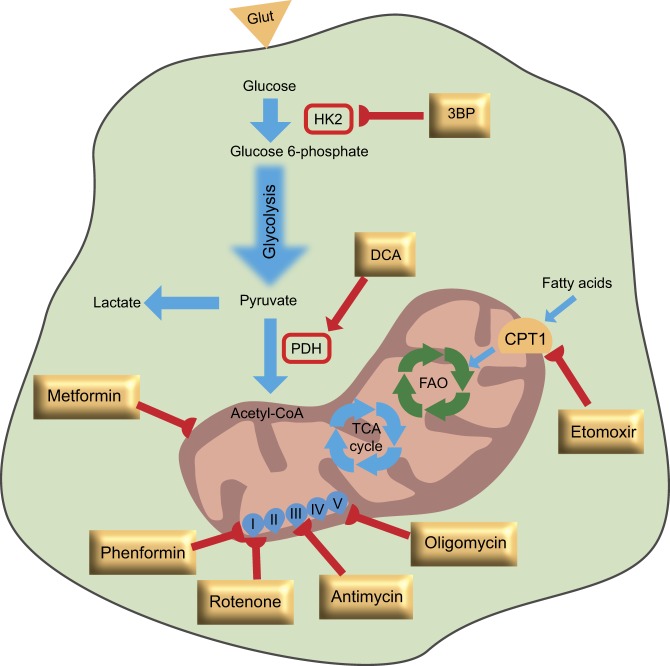
**Metabolic targets of cancer stem cells.** In general, metabolic inhibitors can sensitize cancer stem cells to standard anticancer therapies (highlighted in yellow rectangles), leading to their eradication. Specifically, in models in which cancer stem cells are more reliant on glycolysis, 3-bromopyruvate (3BP) or dichloroacetate (DCA) can reprogram the metabolism of these cells and sensitize them to chemotherapeutic agents. In cancer stem cells that exhibit increased oxidative phosphorylation (OXPHOS), inhibition of mitochondrial respiration by metformin, phenformin, rotenone, oligomycin or antimycin can trigger apoptosis. Inhibition of fatty acid oxidation (FAO) by etomoxir, which inhibits the carnitine-dependent transporter 1 (CPT1), leads to sensitization of cancer stem cells to apoptosis-inducing agents. Glut, glucose transporter; HK2, hexokinase 2; PDH, pyruvate dehydrogenase; I-V, mitochondrial respiratory chain complex I (NADH dehydrogenase subunit), complex II (succinate dehydrogenase subunit), complex III (ubiquinol-cytochrome c reductase complex subunit), complex IV (cytochrome c oxidase subunit) and complex V (ATP synthase subunit).

Several studies using mouse models of cancer have shown that targeting oxidative metabolism, the main source of energy for CSCs in these models, sensitizes this population to chemotherapies, thus leading to their depletion. One example is the population of slow-cycling JARID1B^+^ cells in melanoma that has an upregulation of OXPHOS enzymes. Treatment of melanoma cells with several drugs, including cisplatin and vemurafenib, an inhibitor of mutant BRAF, causes an enrichment of the JARID1B^+^ population and subsequent therapy resistance. When inhibiting OXPHOS using either ATP-synthase inhibitors (oligomycin and Bz-423) or complex I inhibitors (rotenone and phenformin), JARID1B^+^ cells were sensitized to the anticancer agents that initially failed to eliminate them (Fig. 2). Even among functionally and genetically heterogeneous melanomas, this combined approach of treatment was effective, because cytotoxic agents mostly eliminate the rapidly dividing cells, while the metabolic inhibitors could target and sensitize slow-cycling cells (Roesch et al., 2013). Another example is PDAC, where tumourigenesis is essentially driven by mutant *KRAS*. Targeting this oncogene only leads to tumour shrinkage, while sparing a fraction of cells with CSC features that have prominent expression of genes governing mitochondrial function and strong reliance on OXPHOS for cellular energetics. These CSCs showed high sensitivity to OXPHOS inhibitors (preferentially to oligomycin) that, when combined with targeted therapy for the KRAS pathway, could eliminate the tumour and prevent recurrence (Viale et al., 2014). In other studies, however, the mitochondrial inhibitor metformin was not enough to eliminate some CSC clones, possibly due to the heterogeneity and plasticity of PDAC cells and their intermediate glycolytic/respiratory phenotype. These CSC clones showed an upregulation of MYC, which regulates PGC-1α levels and, subsequently, controls the metabolic phenotype of resistant CSC clones. Genetic or pharmacological targeting of MYC reversed this phenotype by increasing the CSC dependency on OXPHOS and sensitizing them to metformin (Sancho et al., 2015) (Fig. 2). Furthermore, the commonly used inhibitor of thymidine synthesis, 5-fluorouracil (5-FU), was shown to selectively target CSCs in colon cancer. These cells undergo a metabolic reprogramming favouring OXPHOS and decreasing the PPP, a mechanism found to be responsible for 5-FU resistance. Thus, combined treatment with 5-FU and metformin abolished drug resistance and effectively diminished the population of CSCs (Denise et al., 2015). In agreement with these findings, treatment of CSCs from epithelial ovarian cancer with different inhibitors of the electron transport chain, namely oligomycin, antimycin and rotenone, could lead to apoptosis of CSCs (Pastò et al., 2014) (Fig. 2).

Targeting glycolytic enzymes also presented promising results in the mesenchymal subtype of glioblastoma CSCs that overexpress ALDH1A3. Radiation treatment of proneural and mesenchymal subtypes of these cells has shown that mesenchymal CSCs are resistant and highly aggressive compared with proneural CSCs. Furthermore, irradiation of proneural CSCs results in an upregulation of mesenchymal-associated markers, and this effect could be attenuated only when inhibiting ALDH1A3. Therefore, the subset of glioblastoma patients with a mesenchymal signature might benefit from ALDH inhibition for the eradication of highly aggressive CSCs (Mao et al., 2013). In another study, the combination of conventional anticancer agents, such as doxorubicin, with a derivative of 3-bromopyruvate that inhibits glycolysis, effectively killed glioblastoma CSCs *in vitro* and inhibited tumour formation *in vivo* (Zhou et al., 2011) (Fig. 2). Furthermore, the metabolic shift from glycolysis to mitochondrial respiration, caused by dichloroacetate treatment, increased ROS and induced apoptosis in glioblastoma CSCs, both *in vitro* and *in vivo* (Michelakis et al., 2010) (Fig. 2).

Lipid metabolism has been also tested as a promising target for the eradication of drug-resistant CSCs. Etomoxir, an inhibitor of the carnitine-dependent transporter CPT1 (also known as CPT1A) and FAO, was able to eradicate ∼50% of quiescent leukaemia SCs in primary human myeloid leukaemia samples, and sensitize leukaemia cells to apoptosis-inducing agents in a murine model (Samudio et al., 2010) (Fig. 2). In hepatocellular carcinoma, the restoration of OXPHOS by Cox6a2 or Cox15 overexpression and/or inhibition of FAO by etomoxir sensitized CSCs to sorafenib treatment, suggesting that reprogramming CSC metabolism by either OXPHOS restoration or FAO inhibition can be an effective therapy in these tumours (Chen et al., 2016) (Fig. 2). Furthermore, slow cycling cells with CSC features and a high capacity to initiate metastasis could be neutralized by antibodies targeting CD36, almost completely inhibiting metastasis in mouse models of oral cancer. These CSCs exhibit high levels of the fatty acid receptor CD36 and lipid metabolism genes, thus providing a novel therapeutic target for the elimination of the metastatic potential of these cells (Pascual et al., 2017).

Another approach for targeting CSC via mitochondrial mass and metabolism is the use of antibiotics, an approach based on the symbiotic theory claiming that mitochondria originated from aerobic bacteria. Several approved antibiotics, such as tetracyclines, salinomycin or erythromycins have been shown to reduce stem properties in cancer, induce differentiation and inhibit tumour growth *in vivo* (Gupta et al., 2009; Zhou et al., 2013)*.*

Collectively, direct targeting of metabolic enzymes or indirect targeting by inhibiting the upstream mediators of metabolic pathways, can lead to CSC eradication in distinct tumour types, mostly in combination with standard anticancer agents (Fig. 2). However, cancer cell plasticity represents a major technical challenge in the design of efficient therapies targeting CSCs. Finally, the notion that by eliminating CSCs, one can achieve cancer cure, requires more reliable and consistent experiments with CSCs in their intact environment, which is still an open field that needs to be addressed.

## Conclusions

The notion that metabolism plays a major role in the maintenance of CSCs is an emerging concept that might provide novel, metabolism-related targets for innovative therapeutic approaches. Although the CSC field has grown exponentially in the last decades, substantial questions remain to be addressed. Specifically, there is an urge to define the mechanisms behind the TME control over the metabolic plasticity of CSCs, and to demonstrate whether CSCs themselves are metabolically heterogeneous and modulate tumourigenesis according to specific stimuli. It is crucial that future studies ameliorate the experimental settings used so far, in order to account for the TME and preserve the *in vivo* structure of tumours.
